# Genetic variants linked to neurodevelopmental disorders within the β3-β4 loop of the TRIO PH2 domain release autoinhibition of GEF2 activity

**DOI:** 10.1016/j.jbc.2025.110429

**Published:** 2025-06-26

**Authors:** Melissa G. Carrizales, Andrew D. Boulton, Anthony J. Koleske

**Affiliations:** 1 Department of Molecular Biophysics and Biochemistry, Yale University, New Haven, Connecticut, USA; 2 Department of Neuroscience, Yale School of Medicine, Yale University, New Haven, Connecticut, USA

**Keywords:** Ras homolog gene family, member A (RhoA), GTPase, guanine nucleotide exchange factor (GEF), neurodevelopment, neurite outgrowth, TRIO family proteins, autoinhibition, PH domain

## Abstract

The TRIO protein contains two guanine exchange factor (GEF) domains, GEF1 and GEF2, which coordinate cytoskeletal rearrangements by activating Rho family GTPases. Rare variants that impact TRIO GEF1 function are associated with autism spectrum disorder, developmental delay, and intellectual disability, but variants are also found throughout the gene. GEF1 promotes GTP exchange on Rac1 and RhoG, while GEF2 activates RhoA. Although GEF1 and GEF2 share a common architecture, the pleckstrin homology (PH) domain in TRIO GEF1 (PH1) assists its activity, while the PH domain in GEF2 (PH2) inhibits its activation of RhoA. A series of single-point variants in the unique **β**3-**β**4 loop of TRIO PH2 has been identified in patients with neurodevelopmental disorders (NDDs), but how they impact TRIO GEF2 activity is not known. Using an *in vitro* fluorescence-based assay to assess GEF2 exchange activity on RhoA, we demonstrate that variants within the **β**3-**β**4 loop relieve GEF2 autoinhibition. Activation of RhoA inhibits neurite outgrowth in Neuro-2A (N2A) cells. GEF2 expression in N2A cells significantly reduces neurite outgrowth, and expression of the G2211E activating GEF2 variant enhances this effect. Together, our findings reveal key interactions and structural constraints for GEF2 autoinhibition and how this mechanism is a target for disruption by NDD-associated variations.

Small GTPases are molecular switches that transition between inactive GDP-bound and active GTP-bound states (1, 2). Active Rho GTPases play diverse roles in cytoskeletal rearrangements in various cell types (3, 4, 5, 6, 7, 8, 9, 10, 11, 12, 13, 14, 15). Guanine nucleotide exchange factors (GEFs) promote GTPase activation by accelerating GDP-to-GTP exchange (7, 16, 17, 18, 19, 20, 21, 22, 23, 24). TRIO, a Rho GEF, can influence multiple Rho GTPases through its two GEF domains (GEF1 and GEF2), suggesting its participation in diverse GTPase pathways (25, 26, 27, 28, 29, 30, 31, 32, 33). GEF1, the most N-terminal TRIO GEF domain, activates the Rac1 and RhoG GTPases, while the more C-terminal GEF2 domain activates RhoA (32, 34, 35, 36). This unique aspect of TRIO is especially interesting given the opposing roles Rac1 and RhoA play in neuronal development in which Rac1 generally promotes neurite growth and stability, while RhoA destabilizes neurites and/or prevents their maturation (3, 10, 34, 37, 38, 39, 40, 41). Rare predicted damaging variants in the *TRIO* gene are linked to autism spectrum disorder (ASD), bipolar disorder (BP), intellectual disability (ID), developmental delay (DD), and schizophrenia (SCZ) and have also been observed in epilepsy (Epi) (37, 42, 43, 44, 45, 46, 47, 48, 49, 50, 51, 52, 53, 54, 55, 56). Understanding how these variants impact TRIO function is key to elucidating the biochemical mechanisms that contribute to these disorders and potentially target these mechanisms for therapy.

While the GEF domains target different substrates, they share a common architecture, each composed of a catalytic Diffuse B cell lymphoma (Dbl) homology domain followed by a regulatory pleckstrin homology (PH) domain (25, 46, 57, 58). Interestingly, the PH domain associated with GEF1 (PH1) promotes GEF1 activity, whereas the GEF2 PH domain (PH2) inhibits GEF2 activity (59). Bandekar *et al.* solved the structure of autoinhibited GEF2, uncovering a new DH–PH interface featuring a unique loop that connects the β3 and β4 sheets in PH2 (Fig. 1, *A* and *B*). This β3-β4 loop extends over the αN helix, which, based on homology comparisons with the closely related p63RhoGEF protein, is predicted to sterically block part of the RhoA binding interface (60). As such, the β3-β4 loop likely makes key contributions to the suppression of GEF2 activity by PH2. Attempts to assess how the removal of the β3-β4 loop impacted activity were hindered by the insolubility of these mutants, so the role of this poorly conserved and disordered loop remains unknown (60). That said, the series of patient-derived variants associated with schizophrenia, bipolar disorder, and epilepsy within the β3-β4 loop of GEF2 suggests its potential in regulating either inter- or intramolecular interactions crucial for TRIO signaling.

**Figure 1:**
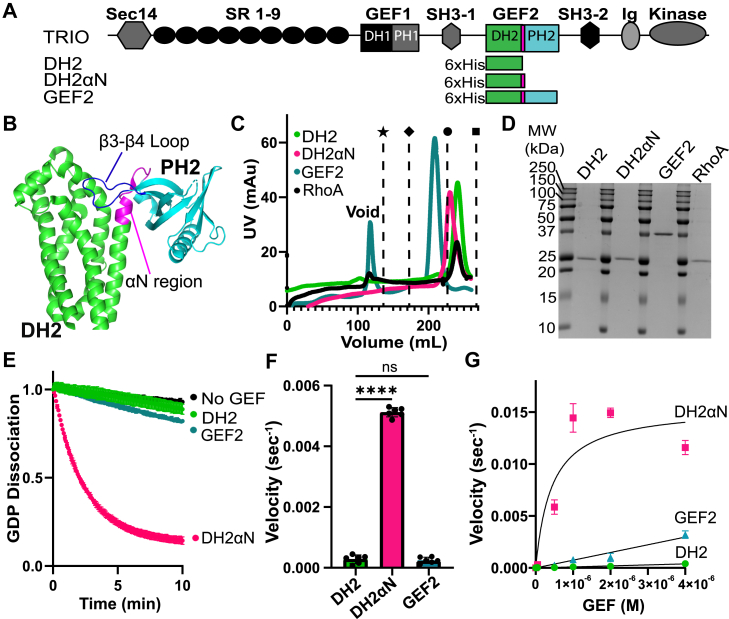
**The GEF2 αN segment promotes GEF activity for RhoA.***A*, schematic of TRIO proteins used in this study: full-length TRIO, DH2, DH2αN, and GEF2; DH, Dbl homology domain; Ig, Ig-like domain; PH, pleckstrin homology domain; SH3, Src homology 3 domains; SR, spectrin repeat. *B*, GEF2 crystal structure with DH2 in *green*, αN in *magenta*, PH2 in *cyan*, and the β3-β4 loop in *dark blue* (PDB 6D8Z). *C*, elution profiles of the indicated proteins from a Superdex 200 Increase 10/300 Gl column. Peak elution positions of commercial protein standards are represented as follows: star = 670 kDa; diamond = 150 kDa; circle = 44.3 kDa; square = 13.7 kDa. *D*, 5 μg of purified proteins were separated by SDS-PAGE and stained with *Coomassie Blue* to assess purity. *E*, 0.5 μM of GEF2 proteins were incubated with RhoA preloaded with 3.2 μM BODIPY-FL-GDP, and GEF activity was measured through the change in fluorescence over time. Curves represent the averages of 6 replicates ± SD. The maximal signal was normalized to 1. *F*, the first minute of activity from panel E was subjected to linear fits to extract the velocity of GDP dissociation for each protein; 0.5 μM DH2 had a 22-fold lower exchange rate than 0.5 μM DH2αN. Activity of the full GEF2 was similar to DH2 alone. N = 6 independent velocity measurements per protein. Mean ± SD; ∗∗∗∗*p* ≤ 0.0001 in a one-way ANOVA adjusted for multiple comparisons; ns = no significance. *G*, Michaelis–Menten plot of velocity as a function of GEF concentration for DH2, DH2αN, and GEF2. Exponential fits to the average of 3 replicates ± SD are shown. DH2 and GEF2 plots did not achieve saturation at the concentrations tested. *The data for DH2αN and GEF2 are also used for comparison in*Figure 2*F*.

Here, we test the hypothesis that the PH2 domain β3-β4 loop is a key regulator of GEF2 activation of RhoA. We present evidence that single neurodevelopmental disorder-associated missense variants within this loop can enhance GEF2 activity on RhoA and inhibit neurite development in mouse neuroblastoma cells. Together, our results clarify how the PH2 domain β3-β4 loop regulates GEF2 activity and the impact of disease-related variants on this regulation. This mechanism may provide a candidate target for therapeutic intervention in the treatment of neurodevelopmental disorders.

## Results

### Maximal TRIO DH2 efficiency requires the **α**N region

Bandekar *et al.* showed the linker region of GEF2 includes a helix they termed the αN helix, which immediately follows DH2 (Fig. 1, *A* and *B*). This helix interacts with the DH2 α helix 3 residues Glu^2069^ and Met^2146^, which are part of the predicted RhoA binding site based on structural homology to RhoA-bound p63RhoGEF (60). To determine whether the αN helix impacts the catalytic activity of the Dbl homology domain 2 (DH2), we purified TRIO DH2 alone (21 kDa) and a DH2+ αN helix residues which links the DH2 and PH2 domains (DH2αN) (22 kDa). We also purified the GEF2 unit (39 kDa), which includes the entire PH2 domain as well as the substrate RhoA GTPase (22 kDa). All proteins eluted at positions consistent with being monomers, as calibrated to commercial protein standards (Sigma-Aldrich, product #69385) (Fig. 1, *C* and *D*).

Catalytic activity of DH2 and DH2αN was measured using a fluorescence-based guanine nucleotide exchange assay, in which RhoA preloaded with fluorescent GDP underwent exchange with unlabeled GTP, resulting in fluorescence decay over time. Purified 0.5 μM DH2 alone catalyzed exchange of fluorescent GDP for GTP on RhoA slowly, with a velocity = 0.000282 ± 0.000142 s^−1^, while the addition of αN accelerated GDP-dissociation by 25-fold (0.00512 ± 0.000153 s^−1^) (Fig. 1, *E* and *F*). To determine the maximum rate of reaction (V_max_), Michaelis constant (K_m_), substrate turnover (k_cat_) and catalytic efficiency (k_cat_/K_m_) of DH2 and DH2αN, we generated Michaelis-Menten plots of velocity as a function of GEF concentration (Fig. 1*G*). While a saturation curve was reached for DH2αN, saturation could not be reached within the concentration range tested for the less active DH2. The addition of αN significantly increased catalysis compared to DH2 (DH2αN k_cat_ = 0.014 ± 0.0013 s^−1^; DH2αN k_cat_/K_m_ = 3.3 × 10^4^ ± 0.619 × 10^4^ M^−1^ s^−1^). These data (summarized in Table 1) indicate that the αN segment is necessary for efficient TRIO GEF2 mediated-GDP/GTP exchange on RhoA.

**Table 1:** Summary of *in vitro* GEF2 nucleotide exchange assays

GEF protein	Velocity (s^−1^)	Fold activation over GEF2 for k_obs_ (s^−1^)	k_cat_ (s^−1^)	K_m_ (M)	k_cat_/K_m_ (M^−1^ s^−1^)
DH2	0.000282 ± 0.000142	NA			
DH2αN	0.00512 ± 0.000153	22	0.014 ± 0.0013	4.25 × 10^−7^ ± 7.82 × 10^−8^	3.3 × 10^4^ ± 0.619 × 10^4^
GEF2_WT_	0.000222 ± 0.000106	NA			
GEF2_WT_	0.0013 ± 0.00017	NA			
GEF2_P2200S_	0.001358 ± 0.000044	NA			
GEF2_L2201H_	0.00155 ± 0.0003201	NA			
GEF2_D2202G_	0.0019 ± 0.00027	1.5			
GEF2_K2204Q_	0.00155 ± 0.00019	NA			
GEF2_P2210L_	0.0018 ± 0.00016	1.4			
GEF2_G2211E_	0.0027 ± 0.00012	2	0.006 ± 0.00012	8.35 × 10^−7^ ± 1.85 × 10^−7^	0.752 ×10^4^ ± 0.137 × 10^4^
GEF2_N2143A/D2144A_	0.000148 ± 0.000056	NA			

0.5 μM RhoA was loaded with BODIPY-FL-GDP in assay buffer, and the reaction was stopped with 5 mM MgCl_2_ after a 1 h incubation. Prior to initiating exchange, 0 to 4 μM TRIO GEF proteins were mixed with 4 mM GTP. Reactions were started by adding the GEF2/GTP mix to the loaded RhoA. Fluorescence was recorded every 10 s for 10 min to monitor BODIPY-FL-GDP release, as described in Blaise *et al.* (61). Fluorescence decay curves were analyzed in GraphPad Prism 10, and the velocity was calculated from the initial slope. Measurements of GEF2 catalytic efficiency were conducted similarly with RhoA concentration fixed at 0.5 μM and increasing concentrations of GEF proteins. Michaelis-Menten plots were created by plotting velocity values *versus* GEF concentration. From this plot, the Michaelis-Menten constant (K_m_) and the catalytic constant, k_cat_, were extracted.

### The TRIO PH2 domain decreases GEF2 catalytic activity on RhoA

While DH domains are sufficient for GEF activity, these activities can be modulated by appended PH domains. Indeed, the TRIO PH2 domain has been reported to inhibit TRIO DH2 domain activity (59). To measure how PH2 impacts catalysis of DH2αN, we generated and purified GEF2, which includes the full DH2αN + PH2 unit (Fig. 1, *A*–*D*). GEF2 showed a 22-fold decrease in catalytic rate (GEF2_WT_ velocity = 0.000222 ± 0.000106 s^−1^) relative to DH2αN (Fig. 1, *E*, *F*, and *G*). Like DH2, GEF2 did not saturate activity in our range of concentration. These observations indicate that the PH2 domain reduces DH2αN-mediated GDP/GTP exchange on RhoA.

### NDD-associated variants within the **β**3-**β**4 loop impact GEF2 catalytic activity

**Figure 2:**
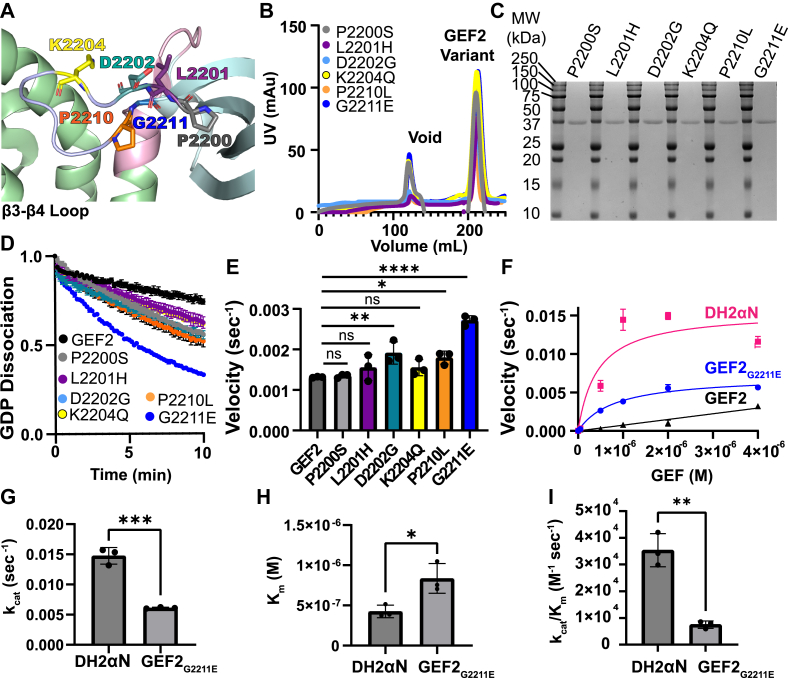
**NDD-associated variants in the PH2 β3-β4 loop release GEF2 autoinhibition.***A*, GEF2 crystal structure (PDB 6D8Z) showing the position of NDD-associated variants within the β3-β4 loop; DH2 domain in *green*; αN in *pink*; PH2 domain in *cyan*; Target residues for variation colored as follows: P2200 in *gray* (BP); L2201 in *purple* (SCZ); D2202 in *teal* (SCZ); K2204 in *yellow* (SCZ); P2210 in *orange* (Epi) and G2211 in *blue* (Epi). *B*, elution profiles of the indicated proteins. *C*, 5 μg of purified components were separated by SDS-PAGE and stained with *Coomassie Blue* to assess purity. *D*, 0.5 μM of GEF2 variant proteins were incubated with RhoA preloaded BODIPY-FL-GDP, and GEF activity was measured by monitoring fluorescence over time. Curves represent the averages of 3 replicates ± SD. *E*, GEF2 P2200S (BP), L2201H (SCZ), and K2204Q (SCZ) did not impact exchange rate relative to GEF2, while D2202G (SCZ), and P2210L and G2211E (both Epi) increased GEF2 activity. N = 3 independent velocity measurements per variant. Bars represent mean ± SD; ∗∗∗∗ = *p* ≤ 0.0001 in a one-way ANOVA adjusted for multiple comparisons. *F*, Michaelis–Menten plot of velocity as a function of GEF concentration for GEF2 and select variants. N = 3 replicates ± SD. GEF2 did not saturate at the concentrations tested. *The data for DH2αN and GEF2 are from*Figure 1*and used here as a reference*. *G*, significant differences were observed for substrate turnover (k_cat_) between DH2αN and the G2211E mutation. Bars represent mean ± SD. N = 3 independent k_cat_ measurements per group. ∗∗∗*p* ≤ 0.001 in a one-way ANOVA adjusted for multiple comparisons. *H*, Significant differences were observed for substrate recognition (K_m_) between DH2αN and the G2211E mutation. N = 3 independent K_m_ measurements per group. Bars represent mean ± SD; ∗*p* ≤ 0.05 in a one-way ANOVA adjusted for multiple comparisons. *I*, significant differences in catalytic efficiency (k_cat_/K_m_) were observed between DH2αN and the G2211E variant. N = 3 independent k_cat_/K_m_ measurements per group. Bars represent mean ± SD; ∗∗*p* ≤ 0.01 in a one-way ANOVA adjusted for multiple comparisons.

We explored variants related to NDDs from the online database Exome Sequencing Meta-analysis (SCHEMA, https://schema.broadinstitute.org/). We found six single-substitution variants near the αN region of GEF2 that are located within the β3-β4 loop of GEF2 in individuals with bipolar disorder, schizophrenia, or epilepsy (Fig. 2*A*). We focused on these variants going forward: P2200S, L2201H, D2202G, K2204Q, P2210L, and G2211E.

We created GEF2 expression constructs containing single NDD-associated point mutations and measured impacts on nucleotide exchange on RhoA (Fig. 2, *B*–*D*). The D2202G (SCZ), and P2210L and G2211E (both Epi) increased GDP dissociation velocity by 1.5-fold, 1.4-fold and 2-fold over WT GEF2, respectively (GEF2_WT_ = 0.0013 ± 0.00017 s^−1^, GEF2_D2202G_ = 0.0019 ± 0.00027 s^−1^, GEF2_P2210L_ = 0.0018 ± 0.00016 s^−1^, GEF2_G2211E_ = 0.0027 ± 0.00012 s^−1^) (Fig. 2*E*).

The maximum catalytic rate could be saturated under the concentration range tested for GEF2_G2211E_ (Fig. 2*F*). We found that the K_m_ was increased approximately 2-fold as compared to active DH2αN (GEF2_G2211E_ K_m_ = 8.35 × 10^−7^ ± 1.85 × 10^−7^ M; DH2αN K_m_ = 4.25 × 10^−7^ ± 7.82 × 10^−8^ M) while GEF2_G2211E_ was 4.7-fold slower than DH2αN in catalytic rate (GEF2_G2211E_ k_cat_ = 0.006 ± 0.00012 s^−1^, k_cat_/K_m_ = 0.752 ×10^4^ ± 0.137 × 10^4^ M^−1^ s^−1^) (Fig. 2, *G*–*I*). These findings are summarized in Table 1 and provide evidence that missense substitutions in the β3-β4 loop of PH2 can relieve autoinhibition.

### G2211E promotes the inhibition of N2A neurite outgrowth by GEF2

**Figure 3:**
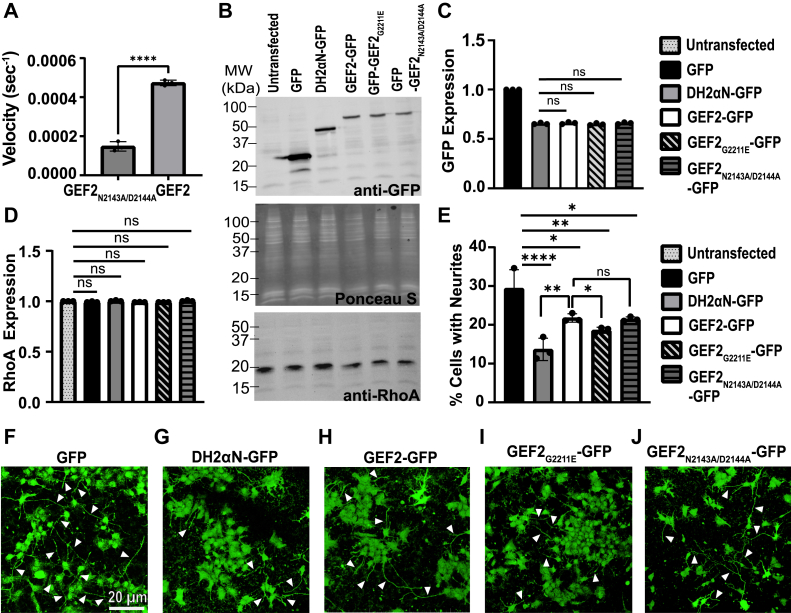
**Activated GEF2 reduces neuritogenesis in N2A Cells.***A*, GEF2_N2143A/D2144A_ and GEF2 proteins were tested with RhoA preloaded with BODIPY-FL-GDP, and GEF activity was measured by monitoring fluorescence over time. N = 3 independent velocity measurements per protein; Mean ± SD; ∗∗∗∗*p* ≤ 0.0001 in a one-way ANOVA adjusted for multiple comparisons. *B* and *C*, expression of each construct was controlled for equal expression; pBlueScriptII SK + DNA was used to maintain DNA levels between all transfections when necessary; N = 3 independent transfections per group; Bars represent mean ± SD; ns = no significance in a one-way ANOVA adjusted for multiple comparisons; GFP signal consistently remained slightly higher; S Ponceau is the loading control. *D*, endogenous RhoA expression was not impacted by transfections; N = 3 independent transfections per group; Bars represent mean ± SD; ns = no significance in a one-way ANOVA adjusted for multiple comparisons. *E*, percentage of cells with neurites from N2A cells; mean ± SD, N = 3 transfections; ∗∗∗∗*p* ≤ 0.0001 in a one-way ANOVA adjusted for multiple comparisons. *F–J*, representative images of N2A cells expressing GFP fusion proteins plated on poly-L-ornithine and laminin-coated cover slips for GFP (*F*), DH2αN-GFP (*G*), GEF2-GFP (*H*), GEF2_G2211E_-GFP (*I*), and GEF2_N2143A/D2144A_-GFP (*J*); *white arrows* point to a neurite.

RhoA activity, through its activation of ROCK kinase, formins, and other effectors, regulates actin assembly and actomyosin contractility. Attenuation of RhoA activity is required for neurite sprouting formation in Neuro-2A (N2A) neuroblastoma cells following serum withdrawal (61). To test how their relative GEF2 activities impacted neurite outgrowth, we expressed GFP fusion proteins, DH2αN-GFP, GEF2-GFP, GEF2_G2211E_-GFP, and a GEF2 mutant we engineered to have reduced catalytic activity, termed GEF2_N2143A/D2144A_ (Fig. 3*A*), in N2A cells at similar levels (Fig. 3, *B* and *C*) and ensured transfection conditions did not alter endogenous RhoA expression (Fig. 3, *B* and *D*). We measured neurites at 48 h after serum starvation (62) (Fig. 3, *E*–*J*).

Although GEF2 is autoinhibited by its PH2 domain, some basal activity remains, and we see this reflected as a 7.7% decrease in neurite outgrowth (GFP control = 29.45 ± 4.7%; GEF2-GFP = 21.8 ± 1.06%). As expected, cells expressing catalytically active proteins, DH2αN and GEF2_G2211E_, had higher reductions of neurite outgrowth by 15.78% and 10.85% respectively (DH2αN-GFP = 13.67 ± 2.8%; GEF2_G2211E_-GFP = 18.6 ± 0.79%). In addition, the reduced activity GEF2_N2143A/D2144A_-GFP inhibited neurite outgrowth to an extent similar to GEF2 (GEF2_N2143A/D2144A_ = 21.5 ± 0.56%). These experiments strongly suggest that the impact of the G2211E variant on GEF2 activity is sufficient to impact the magnitude of a RhoA-dependent process in N2A cells.

## Discussion

Our study reveals fundamental principles and determinants of the catalytic efficiency and regulation of GEF2, a guanine nucleotide exchange factor (GEF) that plays a pivotal role in regulating RhoA activity during neuronal differentiation. The GEF2 domain consists of a DH and PH domain as defined by homology to other DH and PH domains, connected by a unique sequence of residues, including the αN helix. This helix follows the DH2 domain, extending the C-terminal helical structure beyond the canonical DH2 domain. Homology studies with the closely related protein p63RhoGEF suggest that this C-terminal extension contributes to a platform for interactions with RhoA (60). Our findings demonstrate that the inclusion of the αN helix significantly enhances GDP/GTP exchange on RhoA, increasing both the dissociation rate and overall catalytic efficiency, highlighting the essential role of the αN helix in catalysis by GEF2.

We also demonstrate that the TRIO PH2 domain inhibits the catalytic activity of DH2αN. The presence of PH2 reduces the GDP/GTP exchange rate on RhoA by 22-fold, suggesting that PH2 acts as an autoinhibitory regulator. This finding aligns with previous reports showing that PH domains modulate GEF activity (59), and suggests PH2 as a mechanism to control the timing and extent of RhoA activation during neuronal development. The unique role of the PH2 domain in GEF2 regulating RhoA activation is supported by evidence that swapping the PH domain of GEF2 with that of GEF1 increases RhoA exchange activity and reduces Rac1 exchange (59). This inhibitory role may be conserved in some RhoA GEFs, as seen in p63RhoGEF, which carries a GEF domain similar to TRIO GEF2. Previous studies also identified p69RhoGEF as a key Gαq effector crucial for behaviors such as locomotion and egg laying in *Caenorhabditis elegans*, with its function relying on a conserved C-terminal extension of the PH domain, shared with TRIO PH2 (63). While direct interactors of TRIO PH2 are not yet known, it is suggested that PH domains bind lipids, particularly phosphoinositides, which are key components of biological membranes (64, 65, 66). This implies that TRIO localization and its GEF1 and GEF2 activities may be regulated through its two PH domains, potentially within specialized nanodomains in the neuronal membrane, such as PIP3-rich regions in growth cone membranes (67). Increasing evidence reveals how individual PH domains regulate GEF activity in distinct ways, highlighting the diversity in how PH domains control Rho family GTPase signaling.

Variants in TRIO are associated with neurodevelopmental disorders. In TRIO’s GEF2 unit, we identified several disorder-associated variants in the β3-β4 loop of PH2, near the αN helix. Introduction of these variants, found in individuals with bipolar disorder, schizophrenia, and epilepsy, into GEF2 altered nucleotide exchange rates on RhoA, with some mutations significantly increasing the catalytic activity of GEF2. In particular, the G2211E variant exhibited a 2-fold increase in GDP dissociation velocity, indicating that variants in this region at least partly relieve the autoinhibition imposed by the PH2 domain.

**Figure 4:**
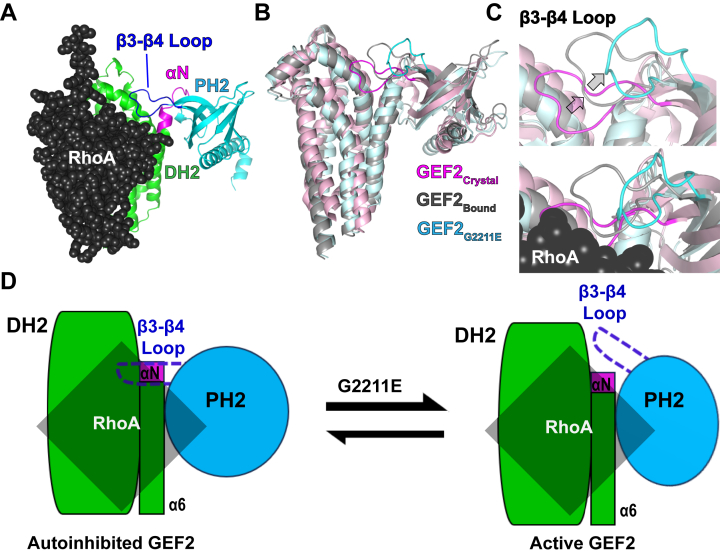
**Alpha Fold 3 predicts a structural shift of the β3-β4 loop in PH2.***A*, the GEF2 crystal structure (PDB 6D8Z) was modeled bound to RhoA in Alpha Fold 3; pLDDT = 90.4; pTM = 0.779. *B*, the unbound, autoinhibited crystal structure (GEF2_crystal_) was aligned to the predicted GEF2:RhoA complex (GEF2_bound_) and the predicted structure for the G2211E mutation (GEF2_G2211E_); RhoA was hidden for clarity. *C*, focused view of the β3-β4 loop from the same alignment as B; RhoA is hidden for clarity in the top panel. *D*, cartoon representation of the structural shift of the β3-β4 loop.

Finally, we explored whether GEF2 can encourage inhibition of neurite outgrowth, a RhoA-dependent process, by assessing neurite extension in N2A cells. The overexpression of various GEF2 constructs, including the G2211E variant, led to reduced neurite extension, with the G2211E variant showing a significant reduction of 10.82%. This suggests that disease-associated mutations in GEF2 not only affect its biochemical activity but also have functional consequences on neuronal morphogenesis. To understand the molecular basis for how the G2211E variant might impact GEF2 catalysis, we utilized AlphaFold 3 (68) to predict its impact on structure (Fig. 4, *A*–*C*). We first modeled GEF2 bound to RhoA (Fig. 4*A*) and aligned it to the crystal structure of free GEF2 (PDB 6D8Z) and the predicted model for the variant GEF2_G2211E_ (Fig. 4*B*). Our analysis revealed that the G2211E variant is predicted to shift the β3-β4 loop, which exposes the αN helix residues for enhanced interactions with RhoA (Fig. 4, *C* and *D*, and Supporting Information Fig. S1) (60). This structural change could explain the increased catalytic activity observed with the G2211E variant, providing insight into how this mutation might disrupt GEF2 regulation and contribute to neurodevelopmental disorders.

Taken together, our findings support a critical role for GEF2 in regulating RhoA during cellular differentiation and suggest that disruptions in its function, through mutations or altered regulatory mechanisms, may contribute to neurodevelopmental disorders. Further studies will be necessary to investigate the broader implications of these mutations *in vivo* and to explore potential therapeutic approaches for modulating GEF2 activity in disease contexts.

## Experimental procedures

### Cloning of expression constructs and protein purification

Human TRIO GEF2 and RhoA constructs were created and affinity-purified from bacterial cells as described (69). Eluted protein was further purified over an S200 Increase column into assay buffer (50 mM Hepes pH 7.25, 150 mM KCl, 5% glycerol, 0.01% Triton, 1 mM DTT) and flash frozen for storage at −80 C. Site-directed mutagenesis was used for point mutations of GEF2. All constructs were confirmed by DNA sequencing. Primers used for cloning are detailed in the Supporting Information.

### GEF2 nucleotide exchange assays

RhoA was loaded with 3.2 μM BODIPY-FL-GDP (Invitrogen) in assay buffer with 2 mM EDTA and incubated for 1 h at room temperature and the loading reaction was halted by adding 5 mM MgCl_2_. Each 30 μl reaction was loaded into 384-well, glass bottom black wall plates (Corning). Prior to initiating the reaction, 0 to 4 μM TRIO GEF2 proteins were combined with 4 mM GTP in a final volume of 10 μl. Exchange reactions were initiated by adding the 10 μl of the GEF2/GTP mix into the 30 μl in the plate wells for a total reaction mixture of 40 μl. Real-time fluorescence data was measured every 10 s for 10 min monitoring BODIPY-FL fluorescence by excitation at 488 nm and emission at 535 nm, as described in Blaise *et al.* (69). Plots of fluorescence decay *versus* time (in seconds) were generated in GraphPad Prism 10 and the velocity of GDP dissociation was determined from the initial slope of the reaction using Equation 1, where Y is the signal for GDP dissociation, and X is time in seconds.

Equation 1 Y=mX+b

Measurements of GEF2 catalytic efficiency were conducted similarly with RhoA concentration fixed at 0.5 μM and increasing concentrations of GEF proteins (0.05 μM, 0.5 μM, 1 μM, 2 μM, and 4 μM). At least three replicates per GEF concentration were performed. Michaelis-Menten plots were created by plotting GDP dissociation velocity value*s versus* GEF concentration. From this plot, the Michaelis-Menten constant (K_m_) was extracted using Equation 2 in GraphPad Prism 10, where K_m_ is in M.

Equation 2 $$Y=V_{\max}*X/\left(K_{m}+X\right)$$

The catalytic constant, k_cat_, values were extracted using Equation 3 below in GraphPad Prism 10, where E_t_ is the GEF concentration. Catalytic specificity was determined by the ratio of k_cat_/K_m_.

Equation 3 $$Y=E_{t}*k_{\text{cat}}*X/\left(K_{m}+X\right)$$

### Transfection of neuroblastoma cells

N2A cells were plated on 6 cm plates with DMEM containing 10% FBS and transfected with 6 μg of plasmid DNA per plate using Lipofectamine 2000 (Invitrogen). After 48 h, cells were used for immunoblot analysis or scored for neurites after serum starvation with DMEM containing 0.5% FBS. Cells that had neurites greater than twice the cell body length were scored as cells with neurites by a reviewer blinded to transfection conditions. 0.5 μg of N1-mCherry was co-transfected to visualize transfection efficiency. pBlueScript II SK + DNA was used to maintain equal DNA levels during transfection when necessary. Images were obtained at 20X magnification using the Dragonfly 630 confocal microscope (Andor).

### Immunoblot analysis

Five micrograms of cell lysate containing each GFP fusion protein was resolved by SDS-PAGE and transferred to nitrocellulose membranes. Protein detection was performed using one of the following (1) Ponceau S staining (2) anti-GFP antibody (Invitrogen, PA1-28664), or (3) anti-RhoA antibody (Invitrogen, MA1-134). Blots were quantified using ImageJ. Protein band intensities were normalized to total protein content as determined by Ponceau S staining. For comparative analysis, chemiluminescent signals were normalized to the GFP control.

### Protein structure predictions

AlphaFold 3 was used to predict the structure of human GEF2 (residues 1960–2292) in complex with human RhoA and to model the structural impact of the G2211E mutation. Predicted models were aligned to the GEF2 crystal structure (PDB: 6D8Z) using PyMOL v2.6 for comparative analysis.

### Rigor and statistical methods

At least three individual replicates for nucleotide exchange assays per GEF protein and concentration were conducted, and results show means ± SD. The rate of decay was extracted *via* a simple linear regression fit to the initial slopes of decay curves in GraphPad Prism 10. A one-way ANOVA was used to determine the statistical significance of rate constants between GEF2, DH2, DH2αN, and GEF2 variants (two-tailed *p*-value < 0.05) and adjusted using Dunnett’s multiple comparisons test.

For cell-based assays, three individual transfections were conducted for DH2αN, GEF2, and GEF2 variants, and results show means ± SD. One-way ANOVA was used to determine the statistical significance of neurite formation between DH2αN, GEF2, and GEF2 variants (two-tailed *p*-value < 0.05) and adjusted using Dunnett’s multiple comparisons test.

## Data availability

Data available upon request. Contact anthony.koleske@yale.edu for more information.

## Supporting information

This article contains supporting information.

## Conflict of interest

The authors declare that they have no conflicts of interest with the contents of this article.

## References

[1] Jaffe A.B., Hall A. (2005). Rho GTPases: biochemistry and biology. Annu. Rev. Cell Dev. Biol..

[2] Song S., Cong W., Zhou S., Shi Y., Dai W., Zhang H. (2019). Small GTPases: structure, biological function and its interaction with nanoparticles. Asian J. Pharm. Sci..

[3] Hall A. (1998). Rho GTPases and the actin cytoskeleton. Science.

[4] Ridley A.J. (2001). Rho GTPases and cell migration. J. Cell Sci..

[5] Nobes C.D., Hall A. (1995). Rho, rac and cdc42 GTPases: regulators of actin structures, cell adhesion and motility. Biochem. Soc. Trans..

[6] Zipkin I.D., Kindt R.M., Kenyon C.J. (1997). Role of a new Rho family member in cell migration and axon guidance in C. elegans. Cell.

[7] Valdivia A., Goicoechea S.M., Awadia S., Zinn A., Garcia-Mata R. (2017). Regulation of circular dorsal ruffles, macropinocytosis, and cell migration by RhoG and its exchange factor, trio. Mol. Biol. Cell.

[8] Spencer A.G., Orita S., Malone C.J., Han M. (2001). A Rho GTPase-mediated pathway is required during P cell migration in Caenorhabditis elegans. Proc. Natl. Acad. Sci. U. S. A..

[9] Gauthier-Rouvière C., Vignal E., Mériane M., Roux P., Montcourier P., Fort P. (1998). RhoG GTPase controls a pathway that independently activates Rac1 and Cdc42Hs. Mol. Biol. Cell.

[10] Govek E.E., Newey S.E., Van Aelst L. (2005). The role of the Rho GTPases in neuronal development. Genes Dev..

[11] Spiering D., Hodgson L. (2011). Dynamics of the Rho-family small GTPases in actin regulation and motility. Cell Adh. Migr..

[12] Hanna S.J., McCoy-Simandle K., Miskolci V., Guo P., Cammer M., Hodgson L. (2017). The role of rho-GTPases and actin polymerization during macrophage tunneling nanotube biogenesis. Sci. Rep..

[13] Filić V., Mijanović L., Putar D., Talajić A., Ćetković H., Weber I. (2021). Regulation of the actin cytoskeleton via rho GTPase signalling in dictyostelium and mammalian cells: a parallel slalom. Cells.

[14] Ridley A.J. (2006). Rho GTPases and actin dynamics in membrane protrusions and vesicle trafficking. Trends Cell Biol..

[15] Bellanger J.M., Astier C., Sardet C., Ohta Y., Stossel T.P., Debant A. (2000). The Rac1- and RhoG-specific GEF domain of Trio targets filamin to remodel cytoskeletal actin. Nat. Cell Biol..

[16] Keller R.K., Standert M. (1992). GTPases. J. Fla. Med. Assoc..

[17] Peurois F., Veyron S., Ferrandez Y., Ladid I., Benabdi S., Zeghouf M. (2017). Characterization of the activation of small GTPases by their GEFs on membranes using artificial membrane tethering. Biochem. J..

[18] Lane J., Martin T.A., Mansel R.E., Jiang W.G. (2008). The expression and prognostic value of the guanine nucleotide exchange factors (GEFs) Trio, Vav1 and TIAM-1 in human breast cancer. Int. Semin. Surg. Oncol..

[19] Kroon J., Heemskerk N., Kalsbeek M.J.T., de Waard V., van Rijssel J., van Buul J.D. (2017). Flow-induced endothelial cell alignment requires the RhoGEF Trio as a scaffold protein to polarize active Rac1 distribution. Mol. Biol. Cell.

[20] Estrach S., Schmidt S., Diriong S., Penna A., Blangy A., Fort P. (2002). The human Rho-GEF Trio and its target GTPase RhoG are involved in the NGF pathway, leading to neurite outgrowth. Curr. Biol..

[21] Yan Y., Eipper B.A., Mains R.E. (2015). Kalirin-9 and Kalirin-12 play essential roles in dendritic outgrowth and branching. Cereb. Cortex.

[22] May V., Schiller M.R., Eipper B.A., Mains R.E. (2002). Kalirin Dbl-homology guanine nucleotide exchange factor 1 domain initiates new axon outgrowths via RhoG-mediated mechanisms. J. Neurosci..

[23] Wu J.H., Fanaroff A.C., Sharma K.C., Smith L.S., Brian L., Eipper B.A. (2013). Kalirin promotes neointimal hyperplasia by activating Rac in smooth muscle cells. Arterioscler. Thromb. Vasc. Biol..

[24] Alam M.R., Johnson R.C., Darlington D.N., Hand T.A., Mains R.E., Eipper B.A. (1997). Kalirin, a cytosolic protein with spectrin-like and GDP/GTP exchange factor-like domains that interacts with peptidylglycine alpha-amidating monooxygenase, an integral membrane peptide-processing enzyme. J. Biol. Chem..

[25] McPherson C.E., Eipper B.A., Mains R.E. (2005). Multiple novel isoforms of Trio are expressed in the developing rat brain. Gene.

[26] Yoshizuka N., Moriuchi R., Mori T., Yamada K., Hasegawa S., Maeda T. (2004). An alternative transcript derived from the Trio locus encodes a guanosine nucleotide exchange factor with mouse cell-transforming potential. J. Biol. Chem..

[27] Bouquier N., Vignal E., Charrasse S., Weill M., Schmidt S., Léonetti J.P. (2009). A cell active chemical GEF inhibitor selectively targets the Trio/RhoG/Rac1 signaling pathway. Chem. Biol..

[28] Skowronek K.R., Guo F., Zheng Y., Nassar N. (2004). The C-terminal basic tail of RhoG assists the guanine nucleotide exchange factor Trio in binding to phospholipids. J. Biol. Chem..

[29] Seipel K., Medley Q.G., Kedersha N.L., Zhang X.A., O'Brien S.P., Serra-Pages C. (1999). Trio amino-terminal guanine nucleotide exchange factor domain expression promotes actin cytoskeleton reorganization, cell migration and anchorage-independent cell growth. J. Cell Sci..

[30] Peng Y.J., He W.Q., Tang J., Tao T., Chen C., Gao Y.Q. (2010). Trio is a key guanine nucleotide exchange factor coordinating regulation of the migration and morphogenesis of granule cells in the developing cerebellum. J. Biol. Chem..

[31] Briançon-Marjollet A., Ghogha A., Nawabi H., Triki I., Auziol C., Fromont S. (2008). Trio mediates netrin-1-induced Rac1 activation in axon outgrowth and guidance. Mol. Cell Biol..

[32] Blangy A., Vignal E., Schmidt S., Debant A., Gauthier-Rouvière C., Fort P. (2000). TrioGEF1 controls Rac- and Cdc42-dependent cell structures through the direct activation of rhoG. J. Cell Sci..

[33] DeGeer J., Boudeau J., Schmidt S., Bedford F., Lamarche-Vane N., Debant A. (2013). Tyrosine phosphorylation of the Rho guanine nucleotide exchange factor Trio regulates netrin-1/DCC-mediated cortical axon outgrowth. Mol. Cell Biol..

[34] Bellanger J.-M., Lazaro J.-B., Diriong S., Fernandez A., Lamb N., Debant A. (1998). The two guanine nucleotide exchange factor domains of Trio link the Rac1 and the RhoA pathways in vivo. Oncogene.

[35] Bandekar S.J., Chen C.-L., Ravala S.K., Cash J.N., Avramova L.V., Zhalnina M.V. (2022). Structural/functional studies of Trio provide insights into its configuration and show that conserved linker elements enhance its activity for Rac1. J. Biol. Chem..

[36] Hart M.J., Eva A., Zangrilli D., Aaronson S.A., Evans T., Cerione R.A. (1994). Cellular transformation and guanine nucleotide exchange activity are catalyzed by a common domain on the dbl oncogene product. J. Biol. Chem..

[37] Barbosa S., Greville-Heygate S., Bonnet M., Godwin A., Fagotto-Kaufmann C., Kajava A.V. (2020). Opposite modulation of RAC1 by mutations in TRIO is associated with distinct, domain-specific neurodevelopmental disorders. Am. J. Hum. Genet..

[38] Sander E.E., ten Klooster J.P., van Delft S., van der Kammen R.A., Collard J.G. (1999). Rac downregulates Rho activity: reciprocal balance between both GTPases determines cellular morphology and migratory behavior. J. Cell Biol..

[39] Yamaguchi Y., Katoh H., Yasui H., Mori K., Negishi M. (2001). RhoA inhibits the Nerve growth factor-induced Rac1 activation through rho-associated kinase-dependent pathway∗. J. Biol. Chem..

[40] Ridley A.J., Hall A. (1992). The small GTP-binding protein rho regulates the assembly of focal adhesions and actin stress fibers in response to growth factors. Cell.

[41] Ridley A.J., Paterson H.F., Johnston C.L., Diekmann D., Hall A. (1992). The small GTP-binding protein rac regulates growth factor-induced membrane ruffling. Cell.

[42] Rao S., Sadybekov A., DeWitt D.C., Lipka J., Katritch V., Herring B.E. (2024). Detection of autism spectrum disorder-related pathogenic trio variants by a novel structure-based approach. Mol. Autism.

[43] Ba W., Yan Y., Reijnders M.R., Schuurs-Hoeijmakers J.H., Feenstra I., Bongers E.M. (2016). TRIO loss of function is associated with mild intellectual disability and affects dendritic branching and synapse function. Hum. Mol. Genet..

[44] Gazdagh G., Hunt D., Gonzalez A.M.C., Rodriguez M.P., Chaudhry A., Madruga M. (2023). Extending the phenotypes associated with gene variants in a cohort of 25 patients and review of the literature. Am. J. Med. Genet. A..

[45] Pengelly R.J., Greville-Heygate S., Schmidt S., Seaby E.G., Jabalameli M.R., Mehta S.G. (2016). Mutations specific to the Rac-GEF domain of TRIO cause intellectual disability and microcephaly. J. Med. Genet..

[46] Tao T.J.S., Min-Sheng Z. (2020). The triple functional domain protein trio with multiple function in the nervous system. J. Neurol. Nueromedicine.

[47] Sadybekov A., Tian C., Arnesano C., Katritch V., Herring B.E. (2017). An autism spectrum disorder-related de novo mutation hotspot discovered in the GEF1 domain of Trio. Nat. Commun..

[48] Schultz-Rogers L., Muthusamy K., Pinto e Vairo F., Klee E.W., Lanpher B. (2020). Novel loss-of-function variants in TRIO are associated with neurodevelopmental disorder: case report. BMC Med. Genet..

[49] Katrancha S.M., Wu Y., Zhu M., Eipper B.A., Koleske A.J., Mains R.E. (2017). Neurodevelopmental disease-associated de novo mutations and rare sequence variants affect TRIO GDP/GTP exchange factor activity. Hum. Mol. Genet..

[50] Liu Y., Liang Z., Cai W., Shao Q., Pan Q. (2022). Case report: phenotype expansion and analysis of TRIO and CNKSR2 variations. Front. Neurol..

[51] Katrancha S.M., Shaw J.E., Zhao A.Y., Myers S.A., Cocco A.R., Jeng A.T. (2019). Trio haploinsufficiency causes neurodevelopmental disease-associated deficits. Cell Rep..

[52] Awasaki T., Saito M., Sone M., Suzuki E., Sakai R., Ito K. (2000). The Drosophila trio plays an essential role in patterning of axons by regulating their directional extension. Neuron.

[53] Varvagiannis K., Vissers L., Baralle D., de Vries B.B.A., Gazdagh G., Adam M.P., Feldman J., Mirzaa G.M., Pagon R.A., Wallace S.E., Amemiya A. (1993). GeneReviews.

[54] Ishchenko Y., Jeng A.T., Feng S., Nottoli T., Manriquez-Rodriguez C., Nguyen K.K. (2025). Heterozygosity for neurodevelopmental disorder-associated TRIO variants yields distinct deficits in behavior, neuronal development, and synaptic transmission in mice. Elife.

[55] Bircher J.E., Corcoran E.E., Lam T.T., Trnka M.J., Koleske A.J. (2022). Autoinhibition of the GEF activity of cytoskeletal regulatory protein Trio is disrupted in neurodevelopmental disorder-related genetic variants. J. Biol. Chem..

[56] Bircher J.E., Koleske A.J. (2021). Trio family proteins as regulators of cell migration and morphogenesis in development and disease - mechanisms and cellular contexts. J. Cell Sci..

[57] van Rijssel J., van Buul J.D. (2012). The many faces of the guanine-nucleotide exchange factor trio. Cell Adh. Migr..

[58] Eid L., Lokmane L., Raju P.K., Tene Tadoum S.B., Jiang X., Toulouse K. (2025). Both GEF domains of the autism and developmental epileptic encephalopathy-associated Trio protein are required for proper tangential migration of GABAergic interneurons. Mol. Psychiatr..

[59] Bellanger J.M., Estrach S., Schmidt S., Briançon-Marjollet A., Zugasti O., Fromont S. (2003). Different regulation of the Trio Dbl-Homology domains by their associated PH domains. Biol. Cell.

[60] Bandekar S.J., Arang N., Tully E.S., Tang B.A., Barton B.L., Li S. (2019). Structure of the C-terminal guanine nucleotide exchange factor module of Trio in an autoinhibited conformation reveals its oncogenic potential. Sci. Signal.

[61] Da Silva J.S., Medina M., Zuliani C., Di Nardo A., Witke W., Dotti C.G. (2003). RhoA/ROCK regulation of neuritogenesis via profilin IIa-mediated control of actin stability. J. Cell Biol..

[62] Lee W.J., Chen L.C., Lin J.H., Cheng T.C., Kuo C.C., Wu C.H. (2019). Melatonin promotes neuroblastoma cell differentiation by activating hyaluronan synthase 3-induced mitophagy. Cancer Med..

[63] Williams S.L., Lutz S., Charlie N.K., Vettel C., Ailion M., Coco C. (2007). Trio's Rho-specific GEF domain is the missing Galpha q effector in C. elegans. Genes Dev..

[64] Harlan J.E., Hajduk P.J., Yoon H.S., Fesik S.W. (1994). Pleckstrin homology domains bind to phosphatidylinositol-4,5-bisphosphate. Nature.

[65] Edlich C., Stier G., Simon B., Sattler M., Muhle-Goll C. (2005). Structure and phosphatidylinositol-(3,4)-bisphosphate binding of the C-terminal PH domain of human pleckstrin. Structure.

[66] Xie M.J., Ishikawa Y., Yagi H., Iguchi T., Oka Y., Kuroda K. (2019). PIP(3)-Phldb2 is crucial for LTP regulating synaptic NMDA and AMPA receptor density and PSD95 turnover. Sci. Rep..

[67] Newsome T.P., Schmidt S., Dietzl G., Keleman K., Åsling B., Debant A. (2000). Trio combines with dock to regulate Pak activity during photoreceptor axon pathfinding in Drosophila. Cell.

[68] Jumper J., Evans R., Pritzel A., Green T., Figurnov M., Ronneberger O. (2021). Highly accurate protein structure prediction with AlphaFold. Nature.

[69] Blaise A.M., Corcoran E.E., Wattenberg E.S., Zhang Y.-L., Cottrell J.R., Koleske A.J. (2021). In vitro fluorescence assay to measure GDP/GTP exchange of guanine nucleotide exchange factors of Rho family GTPases. Biol. Methods Protoc..

