# Supplementary material for: Genetic variants linked to neurodevelopmental disorders within the β3-β4 loop of the TRIO PH2 domain release autoinhibition of GEF2 activity
Source: J Biol Chem. 2025 Jun 26;301(8):110429. doi: 10.1016/j.jbc.2025.110429 (PMC12309612; doi:10.1016/j.jbc.2025.110429)
Supplement: Supplemental Figure 1 [file mmc1.pdf]

Genetic variants linked to neurodevelopmental disorders within the β3-β4 loop of the TRIO PH2 domain release autoinhibition of GEF2 activity.

Melissa G. Carrizales[1], Andrew D. Boulton[1], Anthony J. Koleske[1,2,3]

List of materials included:

1. Supplemental Figure 1

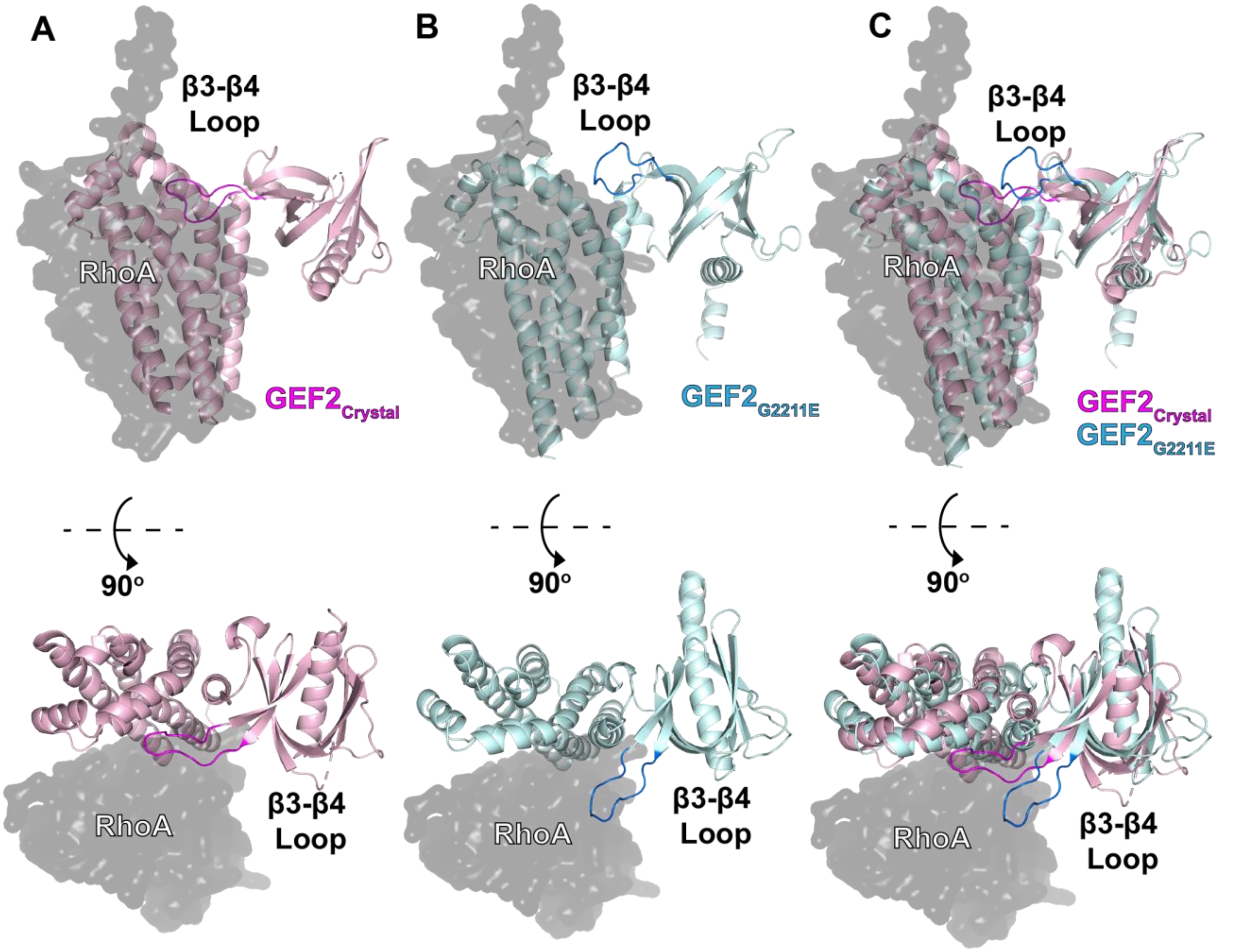

Supplemental Figure 1. Alpha Fold 3 predicts a conformational shift in the β3–β4 loop of PH2. *A,* The crystal structure of GEF2 bound to RhoA (PDB: 6D8Z) shows the β3–β4 loop in a closed conformation. *B,* The predicted structure of the $GEF2_{G2211E}$ mutant bound to RhoA reveals a repositioning of the β3–β4 loop. *C,* Structural alignment of the predicted $GEF2_{G2211E}$ and the crystal structure ($GEF2_{crystal}$) highlights the shift in β3–β4 loop position.
